# Evaluation of measurement uncertainty results calculated by two different methods and total analytical error for ethanol testing

**DOI:** 10.5937/jomb0-55093

**Published:** 2025-07-04

**Authors:** Mehmet Akif Bozdayi, İsmet Gamze Bozdayı, Gökhan Çakırca

**Affiliations:** 1 Mehmet Akif Inan Education and Research Hospital, Department of Medical Biochemistry, Sanliurfa, Türkiye; 2 Sanliurfa Education and Research Hospital, Department of Medical Biochemistry, Sanliurfa, Türkiye

**Keywords:** ethanol, total allowable error, measurement uncertainty, etanol, ukupna dozvoljena gre{ka, merna nesigurnost

## Abstract

**Background:**

The analytical performance metrics of ethanol testing are critically important due to their legal implications when presenting and interpreting results. Measurement uncertainty (MU) and total analytical error (TAE) are essential approaches for evaluating and improving the quality of measurement procedures. This study aimed to calculate MU and TAE values, which assess the reliability of ethanol test results from different perspectives, and to evaluate the impact of MU values, calculated using two different methods, on the legal threshold.

**Methods:**

MU values were calculated following the guidelines of Nordtest and ISO/TS 20914. TAE was determined using the formula TAE%=1.65×CV%+Bias%. External and internal quality data from ethanol testing conducted between July 1, 2022, and June 30, 2024, were used for calculations.

**Results:**

The expanded MU values for ethanol testing were 13.95% according to the Nordtest Guide, 10.94% for low level and 9.59% for high level according to the ISO/TS 20914 Guide. The calculated TAE values were 12.59 for low levels, 11.47 for high levels, and 12.57 overall. MU and TAE values for ethanol testing in our laboratory remained within the allowable total error (±20%) according to CLIA 2024.

**Conclusions:**

We believe each laboratory should report ethanol test results and their respective MU values, particularly when evaluating results close to legal thresholds. Furthermore, we suggest that scientific committees standardise the method for calculating MU and define a target limit.

## Abstract (sr)

**Uvod:**

Metrike analitičkih performansi testiranja etanola od su{tinskog su značaja zbog njihovih zakonskih implikacija pri prezentaciji i interpretaciji rezultata. Merna nesigurnost (MU) i ukupna analitička greška (TAE) su ključni pristupi za ocenu i unapre|enje kvaliteta mernih postupaka. Cilj ove studije bio je izračunavanje MU i TAE vrednosti, koje procenjuju pouzdanost rezultata testiranja etanola iz različitih perspektiva, kao i procena uticaja MU vrednosti izračunatih pomo}u dve različite metode na zakonski prag.

**Metode:**

MU vrednosti izračunate su prema Nordtest smernicama i ISO/TS 20914. TAE je određen pomoću formule TAE%=1,65×CV%+Bias%. Za izračunavanje su korišćeni eksterni i interni podaci o kvalitetu testiranja etanola sprovedenog u periodu od 1. jula 2022. do 30. juna 2024.

**Rezultati:**

Proširene MU vrednosti za testiranje etanola iznosile su 13,95% prema Nordtest smernicama, 10,94% za niske nivoe i 9,59% za visoke nivoe prema smernicama ISO/TS 20914. Izračunate TAE vrednosti bile su 12,59% za niske nivoe, 11,47% za visoke nivoe i 12,57% ukupno. MU i TAE vrednosti za testiranje etanola u našoj laboratoriji ostale su u okviru dozvoljene ukupne greške (±20%) prema CLIA 2024.

**Zaključak:**

Smatramo da bi svaka laboratorija trebalo da pri javljuje rezultate testiranja etanola zajedno sa odgovarajućim MU vrednostima, posebno pri evaluaciji rezultata bliskih zakonskim pragovima vrednosti. Takođe, predlažemo da naučni odbori standardizuju metodu izračunavanja MU i definišu ciljni limit.

## Introduction

Alcohol consumption is associated with various health issues, including mental health disorders, psychosocial dysfunction, liver diseases, and cardiovascular conditions. Moreover, excessive alcohol use not only causes personal harm but is also linked to accidents, such as car crashes, that harm others [1]. Data indicate that approximately 10% of traffic accident fatalities are related to driving under the influence of alcohol. Many countries enforce specific legal limits for blood alcohol concentration (BAC) in drivers. The World Health Organization has established a maximum acceptable BAC of 50 mg/dL for general drivers to address the risk of alcohol-impaired driving [2]. Similarly, in Turkiye, the Road Traffic Act sets the maximum allowable BAC for private vehicle drivers at 50 mg/dL in our country. The relevant law includes various penalties for drivers found to exceed this legal alcohol limit [3]. Given that ethanol test results are categorised as negative or positive regarding legal thresholds, the accurate interpretation of results near these limits is particularly critical. In this context, analytical performance data is important when presenting and interpreting laboratory results [4].

Laboratories aim to meet quality targets set by authorities through quality control studies. One of these targets is Total Allowed Error (TEa). To assess their analytical quality, laboratories compare their TAE with the TEa [5]. All measurements are subject to specific errors. MU provides information on the potential magnitude of these errors [6]. MU is a statistical parameter that indicates the range within which the measured values may vary due to various factors in biochemical measurements [7]. The result obtained from a measurement represents the best estimate of the quantity, and the added uncertainty specifies the range within which the true value is expected to lie at a certain confidence level [8]. MU significantly contributes to the evaluation of test results in clinical practice. When MU is reported alongside test results, end-users can better assess the true representation of the value [4]. Various international guidelines propose different methods for estimating MU [9]. The Nordtest Guide [10] and the ISO/TS 20914 Guide [11] are among the guides commonly used for MU calculations.

Measurement uncertainty and TAE are crucial approaches for evaluating and improving the quality of measurement procedures [12]. This study aimed to calculate MU and TAE values to assess the reliability of etha nol test results from different perspectives and to investigate the impact of MU values, calculated using two different methods (Nordtest and ISO/TS 20914), on laboratory results near the legal threshold (50 mg/dL).

### Ethical declaration

This clinical trial was approved by the Clinical Research Ethics Committee of Harran University (Decision Number: HRÜ/24.13.16, Date: September 09, 2024).

## Materials and methods

### Data acquisition

Our study’s results were retrospectively obtained from the Internal Quality Control (IQC) and External Quality Assessment (EQA) and reported ethanol test reports. In our laboratory, IQC studies are conducted daily by analysing two levels (low and high). IQC studies are performed using control materials provided by the kit manufacturer (Roche Diagnostics, Germany). During the study period (July 1, 2022–June 30, 2024), low-level and high-level IQC analyses were performed using three different lots for each level of control materials (Table 1). The low-level controls had mean±SD values of Lot 1: 50.2±3.4 mg/dL, Lot 2: 49.3±3.3 mg/dL, and Lot 3: 51.1±3.4 mg/dL. The high-level controls had mean±SD values of Lot 1: 148±10 mg/dL, Lot 2: 144±10 mg/dL, and Lot 3: 148±10 mg/dL.

**Table 1:** Internal quality control materials used.

IQC Level	Lot Number	Mean (mg/dL)	SD	Date Range
High Level	51558100	148	10	01/07/2022–22/09/2022
	58788100	144	10	23/09/2022–31/10/2023
	62312800	148	10	01/11/2023–30/06/2024
Low Level	51558000	50.2	3.4	01/07/2022–22/09/2022
	58788000	49.3	3.3	23/09/2022–31/10/2023
	64400400	51.1	3.4	01/11/2023–30/06/2024

External quality assessment was performed monthly over the same period using the Bio-Rad External Quality Assurance Services (EQAS) program, totalling 24 assessments. Additionally, ethanol test results reported over a two-year period (July 1, 2022 - June 30, 2024) were retrieved from the hospital information system. Ethanol analyses were conducted using enzymatic methods on the Roche Cobas 6000 c501 autoanalyser (Hitachi High-Technologies Corporation, Tokyo, Japan) with the manufacturer’s kits (Roche Diagnostics, Germany). In this enzymatic method, ethyl alcohol and NAD^+^ are converted into acetaldehyde and NADH^+^H^+^ using an alcohol dehydrogenase enzyme. The ethanol concentration in the sample is determined using the photometric absorbance change of NADH formed during the reaction. The assay kit data sheet states that the intra-assay precision is between 0.9% and 1.6%, the inter-assay precision is between 1.2% and 2.4%, and the analytical measurement range is between 10.1 and 498 mg/dL in serum/plasma.

### Measurement uncertainty

The expanded measurement uncertainty for the ethanol test was calculated using the Nordtest Guide [10] and the ISO/TS 20914 Guide [11].

### Measurement uncertainty calculation according to the Nordtest guide

### Step 1: Calculating uRw

Two years of IQC data were used to calculate the standard uncertainty for within-laboratory reproducibility (uRw). The coefficient of variation (CV%) was calculated for each control lot level, and using these CV% values, the uRw value was determined by the following formula [13] [14].


(1) uRw=\sqrt{\frac{(n_a-1)xCV_{1}^{2}+(n_b-1)xCV_{2}^{2}+\cdots +(n_i-1)xCV_{i}^{2}}{(n_a+n_b+\cdots +n_i)-n_{periods}}}

n: Number of IQC results

### Step 2: Calculating Bias

Based on our two years of EQA data, the Root Mean Square of the Bias (RMSbias) and the uncertainty of the nominal values (uCref) were obtained, and these values were used to calculate the uncertainty due to (possible) method or laboratory bias (u(bias)).

RMSbias was calculated using the within-group and between-group biases obtained from the EQA data.


(2) RMSbias=\sqrt{\left [ (\text{Within group bias})^{2}+(\text{ Between group bias})^{2} \right ]/2}

Within-group bias=ΣWithin-group bias/n

Between-group bias=ΣBetween-group bias/n

n=Number of EQA results

uCref was calculated using the average CV% values obtained from the EQA data.


(3) uCref=CV \% /\sqrt{n}

n=Number of peer group participants

The u(bias) was calculated using the RMSbias and uCref values.


(4) u(bias)=\sqrt{RMSbias^{2}+uCref^{2}}

### Step 3: Calculating combined standard measurement uncertainty

The combined standard uncertainty (u_c_) components consist of uRw and u(bias).


(5) u_c=\sqrt{uRw^{2}+u(bias)^{2}}

### Measurement uncertainty calculation according to the ISO/TS 20914 guide

According to the ISO guide, the components of the combined standard uncertainty are uRw, the uncertainty from the certified material (calibrator) (ucal), and u(bias). %uRw was obtained from the two-year IQC data (low and high levels). %ucal was provided by the calibrator manufacturer. According to the ISO/TS 20914 guide, u(bias) can be disregarded if the measurement procedure does not show medically significant bias, does not require an in-house calibrator correction factor, and no bias is detected in EQA peer group reports. Since no significant bias was observed in the external quality data, u(bias) was disregarded.


(6) u_c=\sqrt{\% uRw^{2}+ \% ucal^{2}}

### Calculating expanded measurement uncertainty

The expanded measurement uncertainty (U) is obtained by multiplying the combined standard uncertainty from both guides by the coverage factor (k ≈ 2 for 95% confidence interval).


(7) U=u_c * 2

### Total analytical error calculation

The TAE was calculated using the CV% and bias% values with the following formula [5]: Bias% was calculated by using EQA peer group data, and the CV% was calculated by using IQC data.


(8) TAE \% = 1.65 * CV\% + Bias \%

## Results

Based on the two-year IQC and EQA data for ethanol testing, the expanded measurement uncertainty values were found to be 13.95% according to the Nordtest Guide (Table 2) and 10.94% for low level and 9.59% for high level according to the ISO/TS 20914 Guide (Table 3). When evaluated against the legal threshold of 50 mg/dL for general drivers in our country, the legal threshold was determined as 50±6.98 mg/dL based on the Nordtest Guide and 50±5.47 mg/dL based on the ISO/TS 20914 Guide. The calculated TAE values were 12.59 for low level, 11.47 for high level, and 12.57 overall.

**Table 2:** Data for measurement uncertainty components according to the Nordtest Guide and TAE%.

Analyte	uRw	RMSbias	uCref	u(bias)	uc	U%	TAE%
Ethanol	5.43	4.36	0.41	4.38	6.97	13.95	12.57

**Table 3:** Data for measurement uncertainty components according to the ISO/TS 20914 Guide and TAE%.

Analyte	Level	%uRw	%ucal	uc	U%	TAE%
Ethanol	Low level	5.44	0.56	5.47	10.94	12.59
	High level	4.76	0.56	4.79	9.59	11.47

Our emergency laboratory reported six thousand eight hundred seven ethanol results during these two years. Within the threshold range calculated based on the Nordtest Guide uncertainty (43.02-56.98 mg/dL), 42 results were identified. Of these, 18 were greater than 50 mg/dL, while 24 were equal to or less than 50 mg/dL. For the threshold range calculated based on the ISO/TS 20914 Guide uncertainty (44.53–55.47 mg/dL), 31 results were identified. Among these, 14 results were greater than 50 mg/dL, and 17 were equal to or less than 50 mg/dL.

## Discussion

Measurement Uncertainty and TAE are essential tools for assessing the reliability of laboratory results. TAE and MU are metrics for evaluating laboratory analytical quality [12]. Evaluating the TEA with TEa is the most rational way [13]. Although there is no consensus on defining targets for MU, in practice, the TEa value can be used as a quality target [15]. In our study, the calculated MU and TAE values were evaluated according to the quality target (TEa) of Toxicology CLIA 2024 [16], and they were found to remain within the acceptable range (target value±20%).

Even under optimised conditions, the likelihood of obtaining identical results upon repeating any analysis is low. In other words, the outcomes of chemical reactions are essential components of a distribution [17]. MU is a statistical parameter that indicates the range within which measured biochemical values may vary [7]. Various guidelines in the literature outline methods for calculating MU. However, there is no universally accepted guideline for ensuring the comparability of results. For this reason, it is important to publish the calculated MU of tests to allow comparisons between laboratories [17].

Studies reporting MU data for blood ethanol tests are limited [4] [7] [18] [19]. Catak et al. found an MU of 14.2% using the Roche Cobas c501 device with Roche original kits [18]. Erdogan et al. calculated an MU of 13.12% for ethanol testing using a Cobas Integra 800 device with Roche original kits [19]. Ustundag et al. calculated the MU as 19.74% using a Beckman-Coulter AU400 device with Synchron Systems kits [4]. Another study by Bozkaya et al. reported an MU of 5.8% using the Roche Cobas 6000 Modular device with Roche original kits [7]. Across these studies, MU values ranged from 5.8% to 19.74%, with results from studies using devices and kits similar to ours ranging from 5.8% to 14.2%. All these studies employed the Nordtest Guide for MU calculation [4] [7] [18] [19].

In a study by Nurlu et al., the MU for ethanol testing was calculated using two different methods: ISO/TS 20914 (11.55% for level 1 and 9.13% for level 2) and Nordtest (6.62%) [20]. Similar to our study, the analyses were performed using a Roche Cobas 6000 c501 analyser and Roche original kits. In our study, we calculated the MU for ethanol testing provided in our hospital’s emergency laboratory using two different calculation methods. The MU for ethanol testing was calculated as 13.95% according to the Nordtest Guide, 10.94% for low level and 9.59% for high level according to the ISO/TS 20914 Guide.

Several factors contribute to MU, including environmental conditions, operator skill, maintenance frequency, analytical repeatability, calibration frequency, calibrator uncertainty, and reagent stability [14]. These factors may cause differences in MU values between laboratories using the same method, device, and kits. Laboratories can improve error sources by monitoring their MU values at regular intervals.

The Nordtest and ISO/TS 20914 guidelines are widely used for laboratory MU calculation. While the main components in the Nordtest method are *uRW* and *u(bias)*, in ISO/TS 20914, they are *uRW* and *u(cal)*, with bias excluded unless significant. These differences can result in variations in the calculated MU values. In our study, *uRW* was the most influential factor in MU according to ISO/TS 20914, while the contribution of *u(cal)* was minimal (Table 3) and, in the Nordtest calculations, *uRW* had a greater impact on MU than u(bias) (Table 2) consistent with the findings of Nurlu et al. [20].

For the legal threshold of 50 mg/dL in our country, the MU-based threshold was calculated as 50±6.98 mg/dL (Nordtest) and 50±5.47 mg/dL (ISO/TS 20914). Of the reported ethanol results, 0.62% fell within the threshold of 43.02–56.98 mg/dL (Nordtest), and 0.46% fell within 44.53-55.47 mg/dL (ISO/TS 20914). In the study conducted by Bozkaya G. [7] 0.32% of the reported ethanol results for the 50 mg/dL threshold were found to be affected by MU. In the study by Catak et al. [18], 1.76% of the results were reported to be influenced by MU.

Users’ misconception that analytical results are error-free remains prevalent [9]. However, it is a scientific axiom that every measurement contains some degree of error. Therefore, MU and TAE values should be used to evaluate analytical performance and inform decision-making processes. These values can help define »grey zones« of uncertainty around critical decision thresholds [12]. Considering that ethanol results exceeding legal thresholds can lead to judicial consequences, knowing the MU for ethanol testing is more vital than other routine tests [4]. Reporting ethanol results with their MU improves result reliability [18]. However, as demonstrated in our study, differences in MU calculation methods can influence results near decision thresholds to varying degrees, highlighting the need for standardisation by scientific committees.

One of the limitations of our study is the use of manufacturer’s IQC materials in IQC studies. Ideally, the QC material matrix should be the same as the measured patient samples. Manufacturer’s IQC materials can be produced from the same raw materials and based on formulations similar to those of the calibrator. If IQC material is too dissimilar to the patient sample and/or too similar to the calibrator, some changes in method performance may not be detected effectively [21]. The second limitation of our study is using EQA data in the bias calculation. Bias calculated using EQA data often shows matrix-related bias and hides the bias of the actual patient sample [21].

In conclusion, we believe that laboratories should report their MU values alongside ethanol results, particularly for results near legal thresholds, to enhance the reliability of evaluations. As demonstrated in our study, the choice of MU calculation method can significantly influence the calculated values, affecting the number of results influenced by MU near legal thresholds. This highlights the necessity for scientific committees to standardise MU calculation methods.

## Dodatak

### Funding

This study did not receive any external funding.

### Conflict of interest statement

All the authors declare that they have no conflict of interest in this work.

### List of abbreviations

BAC, blood alcohol concentration;<br>CLIA, clinical laboratory improvement amendments;<br>CV, coefficient of variation;<br>EQAS, external quality assurance services;<br>EQA, external quality assessment;<br>IQC, internal quality control;<br>MU, measurement uncertainty;<br>RMSbias, root mean square of the bias;<br>SD, standard deviation;<br>TAE, total analytical error; TEa, total allowable error;<br>U, expanded measurement uncertainty;<br>u(bias), uncertainty due to (possible) method or laboratory bias;<br>uc, combined standard uncertainty;<br>ucal, uncertainty from the certified material (calibrator);<br>uCref, uncertainty of the nominal values;<br>uRw, within-laboratory reproducibility.
